# Cardiac surgery in acute myocardial infarction: crystalloid versus blood cardioplegia – an experimental study

**DOI:** 10.1186/s13019-020-1058-9

**Published:** 2020-01-08

**Authors:** Andreas Boening, Maximilian Hinke, Martina Heep, Kerstin Boengler, Bernd Niemann, Philippe Grieshaber

**Affiliations:** 1 Department of Cardiovascular Surgery, University Hospital Giessen, Rudolf-Buchheim-Str. 7, 35392 Giessen, Germany; 2 Department of Physiology, Justus Liebig University, Giessen, Germany

**Keywords:** Blood cardioplegia, Crystalloid cardioplegia, Myocardial revascularization, Myocardial infarction, Ischemia-reperfusion injury

## Abstract

**Background:**

Because hearts in acute myocardial infarction are often prone to ischemia-reperfusion damage during cardiac surgery, we investigated the influence of intracellular crystalloid cardioplegia solution (CCP) and extracellular blood cardioplegia solution (BCP) on cardiac function, metabolism, and infarct size in a rat heart model of myocardial infarction.

**Methods:**

Following euthanasia, the hearts of 50 rats were quickly excised, cannulated, and inserted into a blood-perfused isolated heart apparatus. A regional myocardial infarction was created in the infarction group (18 hearts) for 120 min; the control group (32 hearts) was not subjected to infarction. In each group, either Buckberg BCP or Bretschneider CCP was administered for an aortic clamping time of 90 min. Functional parameters were recorded during reperfusion: coronary blood flow, left ventricular developed pressure (LVDP) and contractility (dp/dt max). Infarct size was determined by planimetry. The results were compared between the groups using analysis of variance or parametric tests, as appropriate.

**Results:**

Cardiac function after acute myocardial infarction, 90 min of cardioplegic arrest, and 90 min of reperfusion was better preserved with Buckberg BCP than with Bretschneider CCP relative to baseline (BL) values (LVDP 54 ± 11% vs. 9 ± 2.9% [*p* = 0.0062]; dp/dt max. 73 ± 11% vs. 23 ± 2.7% [*p* = 0.0001]), whereas coronary flow was similarly impaired (BCP 55 ± 15%, CCP 63 ± 17% [*p* = 0.99]). The infarct in BCP-treated hearts was smaller (25% of myocardium) and limited to the area of coronary artery ligation, whereas in CCP hearts the infarct was larger (48% of myocardium; *p* = 0.029) and myocardial necrosis was distributed unevenly to the left ventricular wall.

**Conclusions:**

In a rat model of acute myocardial infarction followed by cardioplegic arrest, application of BCP leads to better myocardial recovery than CCP.

## Background

Acute myocardial infarction can be treated by either percutaneous coronary intervention or coronary artery bypass grafting surgery (CABG) [1]. In this setting, the optimal timing of CABG has been a matter of debate for decades. More recent data suggest that immediate surgery is equally safe and possibly associated with improved outcomes compared to delaying surgery for some days after the acute event [2, 3]. If immediate CABG is the method chosen for myocardial revascularization, the procedure is usually carried out using a heart-lung machine [4]. During cardiac procedures, the goal of myocardial protection is to maximize the functional recovery after cardioplegic cardiac arrest and to minimize the extent of ischemia-reperfusion injury. Adequate myocardial protection of infarcted hearts is challenging compared to the setting of non-infarcted hearts due to pre-existing ischemic damage of the cardiomyocytes, intracellular ATP (adenosine tri phosphate) depletion, decrease of cellular pH and changes of the electrolyte distribution [5, 6]. Generally, different cardioplegia solutions are available for inducing cardiac arrest: crystalloid (CCP) or blood-based (BCP) solutions. Bretschneider CCP is an sodium-poor intracellular cardioplegic solution acting through depletion of extracellular sodium. It is usually applied as a single infusion. Contrarily, Buckberg’s BCB is a potassium-rich extracellular cardioplegic solution acting through stabilization of the cell membrane potential between − 35 and − 60 mV. It is usually applied as repeated infusions [7]. Both solutions have been tested experimentally and clinically, but the results even of meta-analyses are conflicting: Zeng et al. 2014 [8] and Guru et al. 2006 [9] showed superiority of BCP (lower myocardial infarction rate, lower rate of low output syndrome, and lower enzyme release), Sa et al. 2012 [10] found no difference between BCP and CCP regarding risk of death, myocardial infarction, and low output syndrome. Likewise, experimental results have shown superiority of BCP [11–15] or of CCP [16]. Thus, although there are many reports comparing CCP and BCP, it is still unclear which can better prevent ischemia-reperfusion damage.

The goal of this study was to compare the influence of CCP and BCP on cardiac function, metabolism, and infarct size in a rat heart model of myocardial infarction.

## Materials and methods

### Experimental model

This study investigated the effect of different two different cardioplegia solutions on isolated infarcted rat hearts in the experimental setting of a blood-perfused Langendorff apparatus. Control hearts underwent the same experimental procedure without induction of a myocardial infarction.

**Fig. 1:**
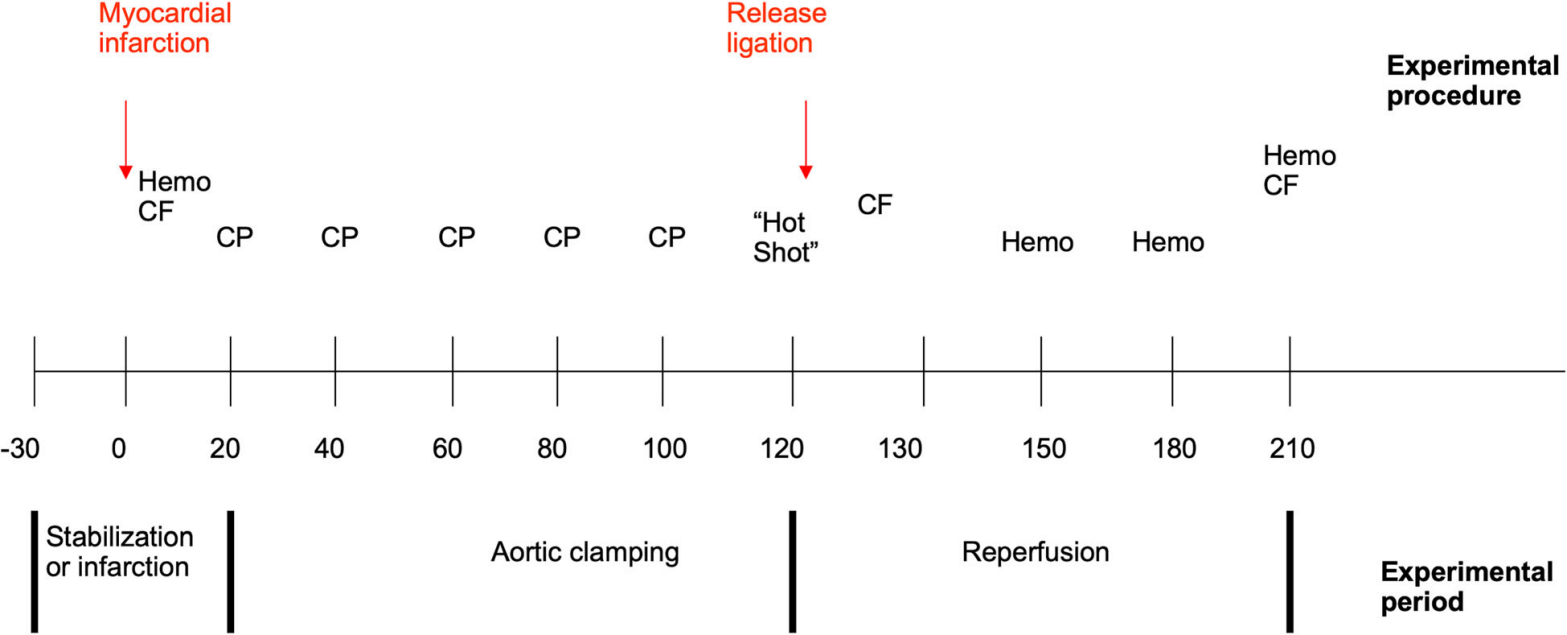
Timeline of the experiment. The timing (in minutes) of actions and interventions for CCP or BCP application in rat hearts subjected to a 120-min myocardial infarction. “Hot shot” refers to warm Buckberg cardioplegia at the end of the clamping time. Abbreviations: CP = cardioplegia, CF = coronary flow, Hemo = hemodynamic measurements

A Langendorff apparatus (Hugo Sachs, Hugstetten, Germany) was filled with freshly prepared, filtered, heparinized bovine erythrocyte concentrates and warmed to a temperature of 36 °C. Physiological conditions were maintained: the hemoglobin content of our perfusion solution was between 6.0 and 7.0 mg/dl. End-diastolic pressure was adjusted to 10–12 mmHg by adding or subtracting volume from the balloon during the first perfusion period of 30 min. After starting the experiment by clamping the aorta, the volume was kept constant. The model has already been described in more detail [17]. After excision of the hearts and during the reperfusion period on the Langendorff apparatus, a myocardial infarction was created in the infarction group by ligation of the left anterior descending coronary artery (LAD) for 120 min. The hearts in the control group did not undergo ligation. After 30 min. of local myocardial inschemia, the aorta was ‘clamped’ for 90 min. Using a three-way-stopcock in the perfusion line. During the 90 min. Clamping time, the hearts were protected with either Bretschneider CCP or Buckberg BCP. The LAD was removed before reperfusing the heart (Fig. 1). To mimic the clinical situation where myocardial protection with BCP and also with CCP is combined with warm (36 °C) extracorporeal circulation, the heart was suspended in a heated (36 °C) chamber. During 90 min of reperfusion, functional parameters were recorded, including coronary blood flow, left ventricular developed pressure (LVDP), and contractility (dp/dt max).

Isovolumetric measurement of left ventricular (LV) performance was carried out using a compliant latex balloon (maximal volume 0.1 ml, 73–2813 IH-SR Balloon Kit for Rats and Guinea Pig Hearts, Hugo Sachs, Hugstetten, Germany) inserted in the LV across the mitral valve that was connected to a pressure transducer. The volume of the saline-filled balloon was kept constant during the entire experiment. LV performance was assessed by measurement of LV systolic pressure (LVSP) and LV end-diastolic pressure (mmHg; LVSP – LV end-diastolic pressure = LVDP). Positive and negative first derivatives of LVSP (+dP/dt and –dP/dt; mmHg/s) were recorded by a transducer-amplifier module (TAM-A, Harvard Apparatus Hugstetten, Germany) and calculated by the software “Isoheart” (Harvard Apparatus). While aortic pressure was held at a constant 70 mmHg by a pressure controller, coronary flow (ml/min) was measured. The results were calculated as percent of baseline to correct for initial differences (Table 1). Baseline hemodynamic measurements were taken after the 30-min stabilization period and before ligature of the LAD or aortic clamping.

**Table 1:** Baseline values of hemodynamic parameters

	Buckberg Infarction (*n* = 9)	Bretschneider Infarction (*n* = 9)	Buckberg No infarction (*n* = 16)	Bretschneide No infarction (*n* = 16)	*p*-Value*
Coronary flow (ml/min)	4 ± 0.3	4 ± 0.6	3 ± 0.3	4 ± 0.4	0.99
LVDP (mmHg)	93 ± 9.5	80 ± 6.5	99 ± 9.3	82 ± 11.0	0.99
dLVP dtmax	2343 ± 228	2430 ± 180	3222 ± 367	2958 ± 321	0.89
dLVP dtmin	− 1946 ± 163	− 1843 ± 110	− 1959 ± 192	− 1838 ± 223	0.88

Values are presented as mean ± standard deviation

Abbreviations: *LVDP* left ventricular developed pressure; *dLVP* derivatives of left ventricular systolic pressure

*P-Value calculated by ANOVA

### Animals and groups

Following euthanasia, the hearts of 50 (age: 3–4 months, weight: 225–350 g) male Wistar rats (Janvier, St. Berthevin, France) were quickly excised, cannulated, and inserted into the blood-perfused isolated heart apparatus. A total of 18 hearts underwent acute myocardial infarction, as described above. Thereof, 9 hearts were protected with cold Buckberg BCP and 9 with cold Bretschneider CCP. A total of 32 hearts served as control groups with 16 hearts in the cold Buckberg BCP group and 16 hearts in the cold Bretschneider CCP group, respectively. All experiments were approved by the regional authorities and conformed to the German Animal Protection Law.

### Cardioplegia

Cold Buckberg BCP was administered in a 4:1 (blood:BCP) dilution. BCP was prepared using the bovine erythrocyte solution used for perfusion of the hearts and a commercially available solution (Köhler Chemie, Bensheim, Germany; [Table 2]). The BCP application flow rate was calculated as the amount of baseline coronary flow + 30% (in ml/min); At this flow rate, cold induction and hot shot were applied over 4 min each, the repeated doses every 20 min over 2 min each. After having measured coronary flow under baseline conditions (Fig. 1), we added a 30% safety margin to the baseline flow to ensure that sufficient BCP was administered. The single dose of cold CCP consisted of 30 ml Bretschneider CCP (Köhler Chemie, Bensheim, Germany) applied under 50–70 mmHg aortic pressure (corresponding to the usually clinically used 120-140 mbar hydrostatic perfusion pressure). The amount of Bretschneider CCP was calculated according to the manufacturer’s instruction for use assuming an estimated heart weight of 4 g (1 ml CCP per minute and gram heart weight over 8 min resulting in 32 ml CCP).

**Table 2:** Composition of blood cardioplegia (crystalloid components)

Components Buckberg	Concentration (mmol/l)	Components Bretschneider	Concentration (mmol/l)
K^+^	I: 17.5 II: 6.1 III: 7.4	KCl^−^	10
Na^+^	I: 8.1 II: 8.4 III: 7.8	NaCl	18
Cl^−^	I: 22.3 II: 13.8 III: 14.2	MgSO_4_	4
Na^+^ glutamate	I: 0 II: 0 III: 14.1	CaCl_2_	0.02
Na^+^ aspartate	I: 0 II: 0 III: 13.9	Mannitol	33
Trometamole	I: 8.7 II: 9.1 III: 13.3	Histidine	180
Citric acid x H_2_O	I: 0.9 II: 0.9 III: 3.5	Histidine HCl	18
Na^+^ citrate x H_2_O	I: 5.2 II: 5.5 III: 19.8	Tryptophan	2
NaH_2_PO_4_ x 2H_2_O	I: 0.9 II: 0.9 III: 3.6	Alpha-ketoglutarate	1
Glucose x H_2_O	I: 184.8 II: 193.9 III: 204.5		

Buckberg solution I was applied for cold induction, solution II for cold reinfusion, and solution III for the “hot shot”. For Bretschneider solution application, see text. The concentrations are given for the ready-to-use mixture of blood and cardioplegia (Buckberg: blood:cardioplegia 4:1)

### Infarct size planimetry

After 90 min of reperfusion, the hearts of the infarction group were removed from the perfusion apparatus and frozen at − 20 °C for 30 min. Subsequently, hearts were cut into 7–8 slices and incubated in 1.2% triphenyl tetrazolium chloride for 20 min at 37 °C. Heart slices were then fixed in 7% formalin at room temperature overnight. Digital images were taken of both sides of the heart slices with a M60 microscope (Leica, Wetzlar, Germany) at 2.5-fold magnification. Infarct size was determined by planimetry using the Leica Application Suite LAS version 4.6 (Leica).

### Statistical analysis

Hemodynamic parameters obtained at different time points after the end of cardioplegia or opening of the aortic clamp were expressed relative to baseline (BL) and analyzed using SPSS statistical software version 24 (IBM, Ehningen, Germany). Intergroup differences in hemodynamic parameters and results of cardiac metabolism (response variables) over time were analyzed using two-way ANOVA (analysis of variance) models with “time points” (categorical) as the within-subject factor and “group” (categorical: “CCP infarction” “BCP infarction”, “CCP non-infarction”, and “BCP non-infarction”) as the between-subjects factor. Tukey’s post-hoc test was used if any difference was observed. The infarct sizes were compared between the groups using a two-sided Student’s t-test. Data are shown as mean ± SD. Statistical significance was assumed at a level of *p* < 0.05.

## Results

**Fig. 2:**
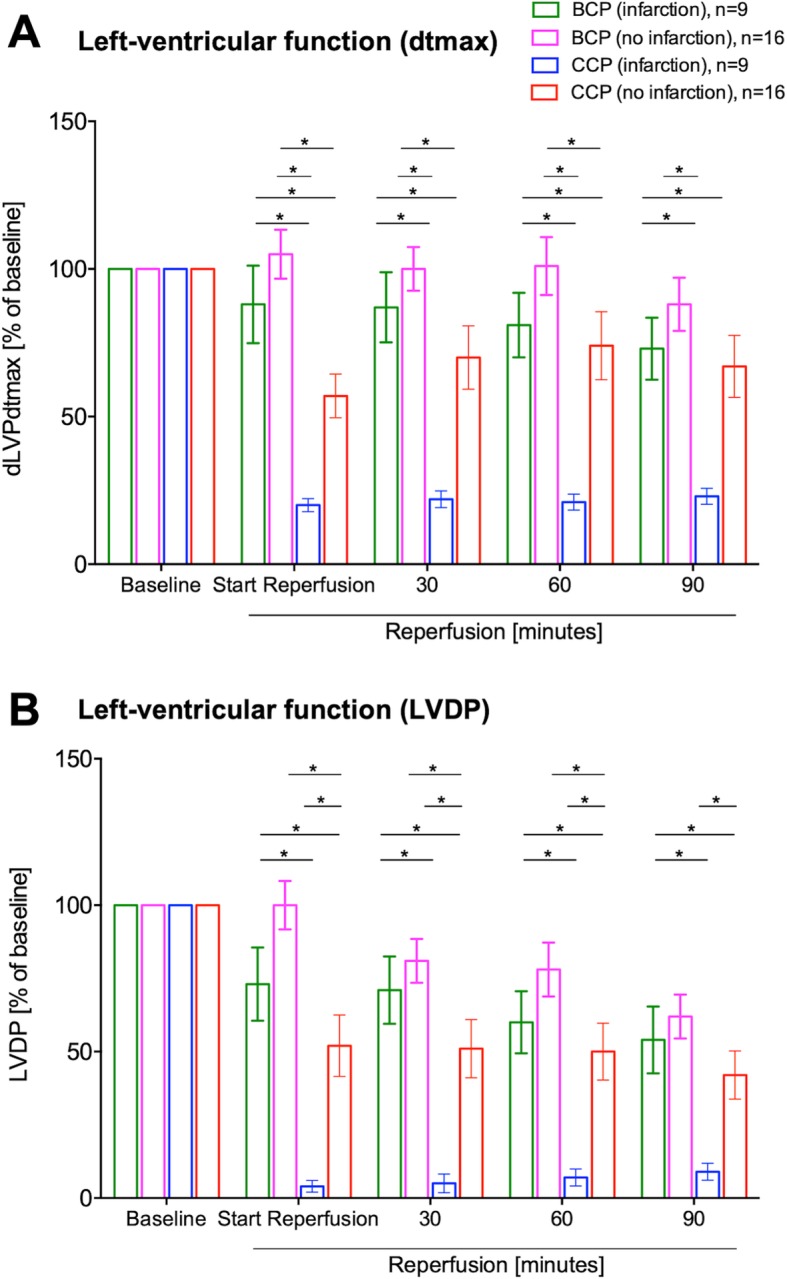
Hemodynamic recovery of cardiac function. Recovery was significantly impaired after application of Bretschneider CCP. Shown are results for dLVPdt_max_ (2 A) and left ventricular developed pressure (LVDP, 2 B).*: p < 0.05. Abbreviations: BCP = blood cardioplegia solution, CCP = crystalloid cardioplegia solution, dLVPdt_max_ = derivatives of left ventricular systolic pressure, LVDP = left-ventricular developed pressure

**Fig. 3:**
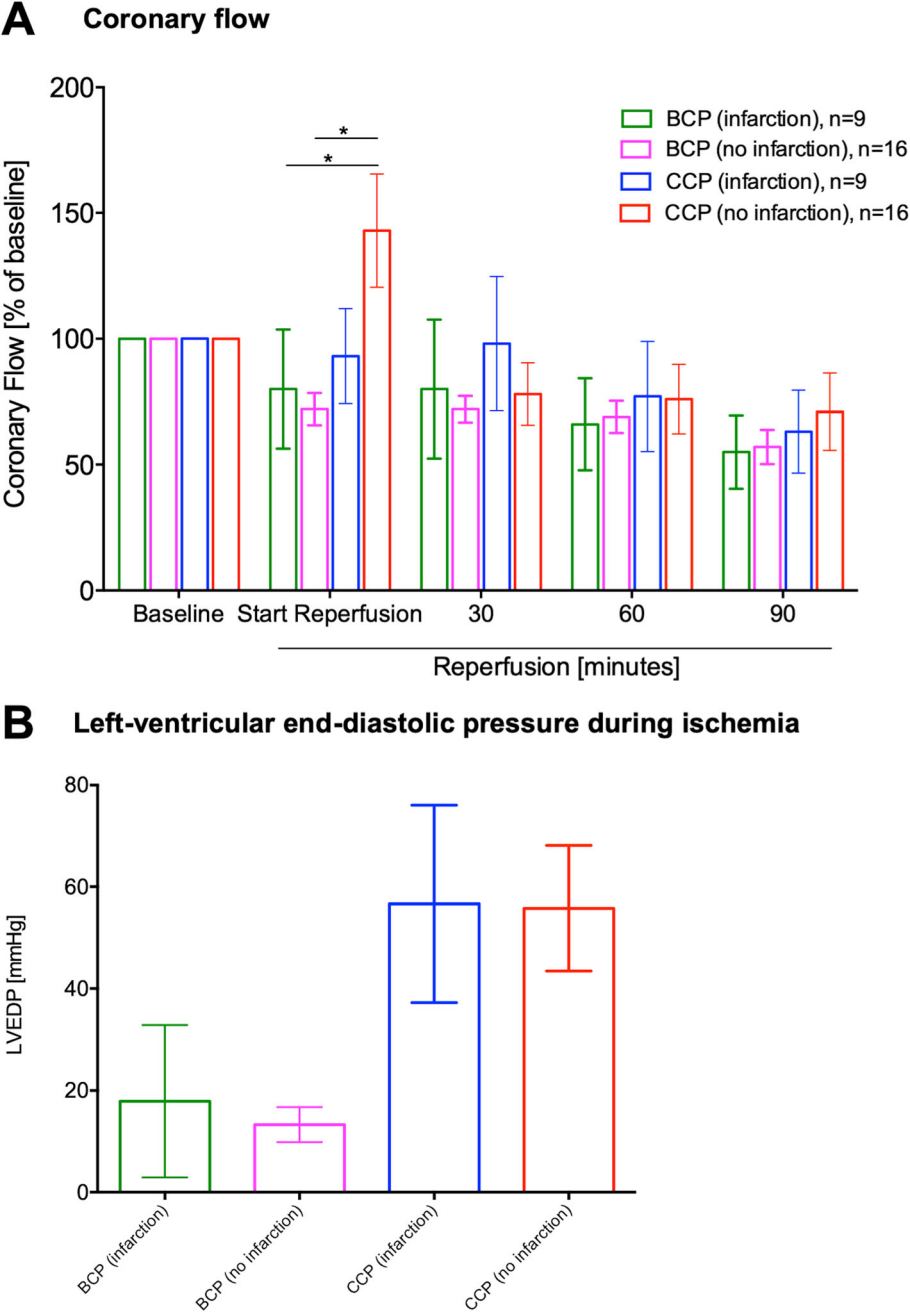
Coronary flow and left ventricular end-diastolic pressure. Coronary flow was similar after Bretschneider CCP or Calafiore BCP (3 A). Left-ventricular end-diastolic pressure (LVEDP) rose during the ischemic period only in CCP hearts (3b).*: *p* < 0.05. Abbreviations: BCP = blood cardioplegia solution, CCP = crystalloid cardioplegia solution, LVEDP = left-ventricular end-diastolic pressure

Cardiac function after acute myocardial infarction and 90 min of cardioplegic arrest was better preserved with Buckberg BCP than with Bretschneider CCP (LVDP 54 ± 11% 9 ± 2.9% [*p* = 0.0062]]; dp/dt max. 73 ± 11% vs. 23 ± 2.7% [*p* = 0.0001]; Fig. 2). In contrast, coronary flow was similarly impaired (BCP 55 ± 15%, CCP 63 ± 17% [*p* = 0.99]) in both types of cardioplegia (Fig. 3). In hearts without infarction, myocardial function was equally preserved after Buckberg and after Bretschneider cardioplegia (LVDP 62 ± 8% vs. 42 ± 8% [*p* = 0.21], dp/dt max. 88 ± 9% vs. 67 ± 11% [*p* = 0.21]).

**Fig. 4:**
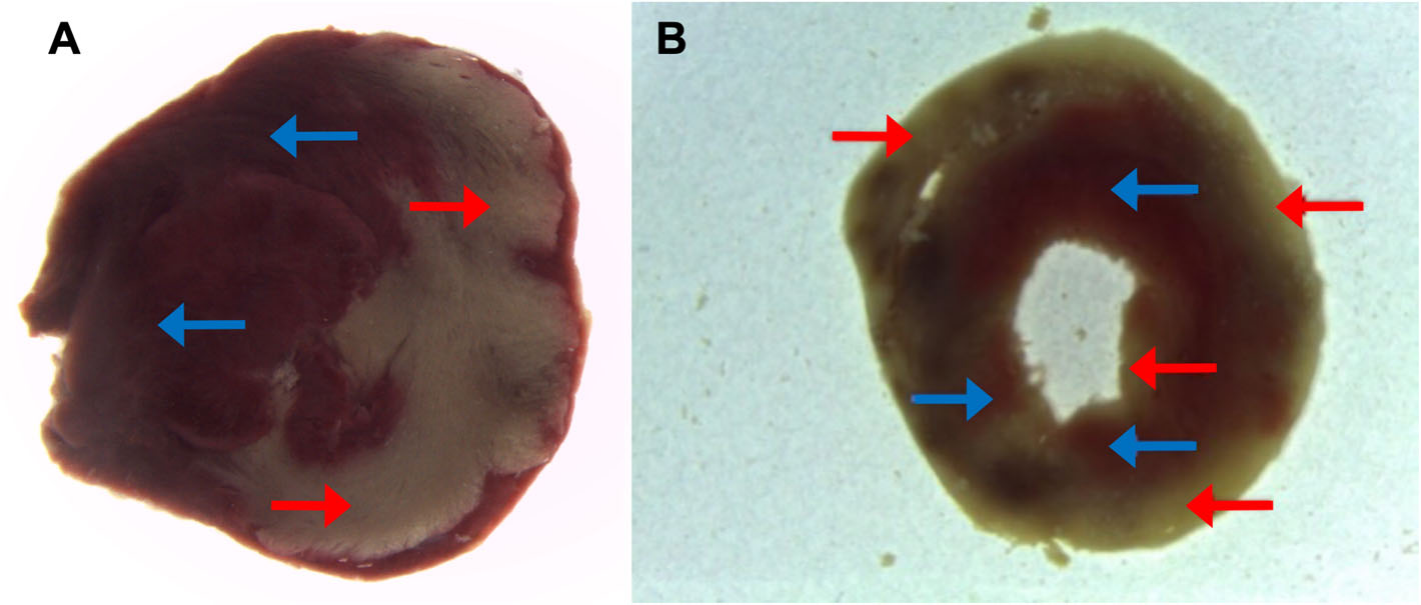
Infarcted areas after ligation. Size of the myocardial infarction in slices of rat hearts after LAD ligation for 120 min, aortic clamping with Bretschneider or Buckberg cardioplegia for 90 min, and reperfusion for 90 min. A) Typical appearance of infarcted area (pale tissue, red arrow) compared with non-infarcted area (red tissue, blue arrow) after coronary artery ligation (Buckberg cardioplegia). B) Differential pattern of infarcted areas after Bretschneider cardioplegia with ischemic areas unevenly distributed in the myocardium

The infarct pattern was different between hearts perfused with BCP or CCP. With BCP, the infarct was small (25% of myocardium) and limited to the area of LAD ligation, whereas with CCP, the infarct was larger (48% of myocardium; *p* = 0.029) and myocardial necrosis was distributed unevenly to the left ventricular wall (Fig. 4). Because the infarct pattern in the CCP hearts resembled subendocardial damage that can be caused by high diastolic LV pressure, we calculated the left-ventricular end-diastolic pressure (LVEDP) during the aortic clamping period. LVEDP rose continuously during the ischemia period after CCP (Fig. 3), whereas it did not rise during the BCP cardioplegia intervals of 20 min. This difference in reaction after CCP versus BCP was also observed in the infarcted (peak_(CCP)_ 57 mmHg vs. peak_(BCP)_ 18 mmHg; *p* = 0.0002) and in the control (peak_(CCP)_ 56 mmHg vs. peak_(BCP)_ 13 mmHg; *p* = 0.0001) hearts.

## Discussion

In our isolated rat heart experiments with an infarction model, we found that cold BCP was superior to cold CCP. Recovery of cardiac function after cardiac arrest was better after BCP, and the infarct size was smaller. Our results support the previous findings of Münch et al. who observed superior contractility after ischemia with BCP in a piglet model [18], Feng et al. who reported less apoptosis, preserved left ventricular function, and lower coronary resistance after BCP in a rabbit model [11], and Runge et al. who found more rapid normalization of myocardial metabolism in a porcine model [12]; all of these experiments were conducted without myocardial infarction. Julia et al. [13], Vinten-Johansen [14], and Catinella et al. [15] showed superiority of BCP over CCP, with better recovery of contractile function after BCP in dog heart infarction models. Only Coetzee et al. [16] showed superiority of CCP compared with BCP due to better functional recovery of the hearts in a baboon model.

As there have been many different models with different species investigated with or without infarction and with completely different cardioplegia compositions, the results are difficult to compare. Several articles compare self-made cardioplegia formulations that are different from the clinically used and experimentally tested Buckberg BCP solution. Buckberg himself has emphasized several times [19, 20] that “blood cardioplegia is not blood cardioplegia”, meaning that changes in composition application and temperature of cardioplegia should be tested first before they are applied in patients. We reported previously in a similar experimental setting that cold Buckberg BCP and warm Calafiore BCP lead to similar functional recovery in the infarcted isolated rat heart. However, the LVEDP rose more in the warm BCP group, possibly suggesting a discrete advantage of cold Buckberg BCP in the setting of AMI [21].

In clinical studies, endpoints like mortality, rate of myocardial infarction, and the occurrence of low-output syndrome are often reported. The problem with these outcome parameters – even in randomized studies or in “best evidence” papers [22] – is that they are influenced by several factors other than cardioplegia (e.g. surgical skills, anesthesia regimen, catecholamines, anticoagulation, cardiopulmonary bypass strategy, severity of coronary artery disease), which leads to a limited effect of cardioplegia on these outcome parameters. This is visible in the fact that three meta-analyses containing up to 36 randomized studies and more than 5500 patients come to conflicting results regarding the efficacy of BCP and CCP on clinical endpoints [8–10]. The routine approach in our daily practice is to apply BCP for all adult cardiac surgery procedures. For patients with acute myocardial infarction or with long expected clamping time, we use Buckberg cold BCP. For cases without myocardial infarction and short expected clamping time of < 90 min., we use Calafiore’s warm BCP. This approach seems to be justified by the present experimental data.

In our study, the difference between BCP and CCP regarding myocardial recovery after myocardial infarction and ischemia/reperfusion was greater than we expected, with nearly no recovery at all after CCP. The recovery was even less compared with previous experiments with a blood-perfused Langendorff apparatus where hearts were subjected to a comparable ischemia-reperfusion setting without application of cardioplegic solution [23]. We also observed better recovery after CCP application in a crystalloid-perfused Langendorff environment [24]. The reduced recovery after CCP application can probably be attributed to the development of cardiac rigor during the clamping period after CCP. At the end of CCP perfusion, mean LVDP was over 60 mmHg, whereas after repeated BCP applications no rigor was observed. Furthermore, in BCP-perfused hearts the infarcted area was limited to the LAD-dependent myocardium, whereas in CCP-perfused hearts it had spread to the whole heart with an emphasis on the subendocardial “inner” myocardial zone of the left ventricle. Another possibly relevant aspect might be calcium-depletion-induced cellular damage (calcium paradox) which has been described as a side-effect of calcium-free cardioplegic solutions [25]. This effect might be of particular relevance in isolated hearts due to the lack of non-coronary collateral flow [26–28].

Another relevant aspect could be the rise of myocardial temperature during the aortic clamping period. In our experiments, we tried to mimic today’s clinical realities: Myocardial temperature was allowed to rise and was not measured during the aortic clamping period. Even in patients with Bretschneider cardioplegia and and additional ice water around the heart, the myocardial temperature rises during the clamping period. If this alone would lead to contracture of the heart, we would see a lot of contracted hearts in our patients treated with Bretschneider cardioplegia, because most surgeons do not allow body temperature to fall below 33 °C or do actively cool not below 28 °C. Thus, we don’t think that rewarming is an important issue leading to the results we have seen.

### Limitations

Although we have tried to mimic the clinical setting regarding temperature, application, and composition of both types of cardioplegia, our experimental setting in an isolated rat heart model is artificial. Moreover, the myocardial infarction is created in a beating and perfused heart for 120 min and not a working heart over a longer period of time. The infarct size of the rat hearts could not be measured before reperfusion. The total number of animals used was rather small. There was only one clamping time (90 min.) investigated in this setting. The effects of other (shorter or longer) clamping times on the efficiency of different protection strategies (eg. development of infarct size) remain to be investigated in further experiments. In addition, we used a buffered bovine erythrocyte suspension in our model instead of whole rat blood, and we cannot be sure how this influenced our findings. Furthermore, cardioplegia delivery to the infarcted LAD area was probably impaired due to the LAD ligature. The extent of collaterals could not be evaluated individually. Therefore, the real protection of the LAD territory achieved during the experiment remains somewhat elusive. However, we chose this model in order to simulate the clinical setting, where delivery of cardioplegia to the infarct area can also be difficult. However, clinically used approaches to deliver cardioplegia to the infarct area (retrograde application, application via bypass grafts) could not be incorporated in this experimental model.

Despite these limitations, no other experimental model comes closer to the clinical setting. With our model, we were able to exclude patient characteristics that can influence the findings in clinical settings. Moreover, the endpoints used in the clinical setting are often too general or too noninvasive to be able to observe slight differences and changes in the myocardium of patients.

## Conclusion

In the investigated isolated rat heart model of acute myocardial infarction followed by cardioplegic arrest, BCP leads to better myocardial recovery than CCP which may be considered in the clinical use of surgical revascularization in patients with acute myocardial infarction.

## Abbreviations

ANOVA: Analysis of variance
BCP: Blood cardioplegia solution
BL: Baseline
CABG: Coronary artery bypass grafting surgery
CCP: Crystalloid cardioplegia solution
LVDP: Left-ventricular developed pressure
LVEDP: Left-ventricular end-diastolic pressure
